# A qualitative analysis of healthcare professionals’ experiences with an internet-based emotion regulation intervention added to acute psychiatric inpatient care

**DOI:** 10.1186/s12888-024-06365-z

**Published:** 2024-12-27

**Authors:** Laura Luisa Bielinski, Gwendolyn Wälchli, Anna Lange, Elianne von Känel, Lena Katharina Demel, Christoph Nissen, Franz Moggi, Thomas Berger

**Affiliations:** 1 Department of Clinical Psychology and Psychotherapy, Institute of Psychology, University of Bern, Fabrikstrasse 8, Bern, 3012 Switzerland; 2 Institute of Social and Preventive Medicine (ISPM), University of Bern, Bern, Switzerland; 3 University Hospital of Psychiatry and Psychotherapy, University of Bern, Bern, Switzerland; 4 Division of Psychiatric Specialties, Geneva University Hospitals, Geneva, Switzerland

**Keywords:** Acute psychiatric inpatient care, Emotion regulation, Healthcare professionals’ experiences, Internet-based intervention

## Abstract

**Background:**

Healthcare professionals play an important role in successfully implementing digital interventions in routine mental healthcare settings. While a larger body of research has focused on the experiences of mental healthcare professionals with the combination of digital interventions and face-to-face outpatient treatment, comparatively little is known about their experiences with digital interventions combined with inpatient treatment. This is especially true for acute psychiatric inpatient care, where studies on the implementation of digital interventions are more rare. The current study aimed to investigate healthcare professionals’ experiences with an internet-based emotion regulation intervention added to acute psychiatric inpatient treatment.

**Methods:**

Physicians, nurses, psychologists, social workers, and occupational therapists from three acute inpatient wards (*n* = 20) were interviewed regarding their experiences. A thematic analysis approach was used to analyze the interview data.

**Results:**

The following themes and corresponding *subthemes* were identified: lack of experience (*few or no previous experiences*,* no expectations*,* few points of contact*), the intervention as a contemporary complement (*positive expectations*,* necessary and contemporary*,* positive effects on therapeutic work and patients*,* characteristics of the internet-based program*), concerns about fit for acute psychiatric inpatient care (*fit for acute psychiatric inpatients*,* doubts about implementation*), the human factor as essential for implementation (*the team makes or breaks it*,* guidance is key*,* patient characteristics*), and requirements for implementation beyond the human factor (*integration into existing treatment structure*,* resources*,* changes to the internet-based program*,* timing*).

**Conclusions:**

While healthcare professionals reported few points of contact with the intervention, they saw it as a contemporary complement to acute psychiatric inpatient care with benefits for therapeutic work and patients. The findings further suggest that specific concerns regarding the fit for acute psychiatric inpatient care remain and that human factors such as support from the ward team, human guidance during the intervention and being mindful of specific patient characteristics are considered important for implementation. Moreover, factors such as integration of the intervention into the ward program, resource availability and the timing of the intervention during a patient’s individual stay should be considered for successful implementation.

**Trial registration:**

Clinicaltrials.gov, NCT04990674, 04/08/2021.

**Supplementary Information:**

The online version contains supplementary material available at 10.1186/s12888-024-06365-z.

## Background

Healthcare professionals play a pivotal role in the delivery of mental healthcare interventions in routine care settings. For integrating digital interventions, they are considered relevant and understudied stakeholders [1, 2]. Mental healthcare professionals’ attitudes may be more favorable toward the combination of digital interventions with face-to-face (f2f) treatment than toward standalone digital interventions [3]. When digital interventions are combined with f2f treatment, this is termed blended treatment, blended care or blended therapy (BT). BT can take on many different forms. Examples include the concurrent application of an internet-based intervention and f2f treatment, and sequential applications where an internet-based intervention is provided, for example, as aftercare or prior to face-to-face therapy [4, 5]. Several studies have reported on the effectiveness of BT for mental health disorders with the majority of studies focusing on the outpatient setting and the treatment of depressive symptoms [5, 6]. Recent reviews have also provided evidence for the feasibility of BT for the treatment of severe mental health disorders [7] and for the effectiveness of BT in inpatient settings [8].

The experiences of mental healthcare professionals with BT have been examined for different treatment settings. Several studies examined experiences in the outpatient mental healthcare setting. For example, Titzler et al. [9] examined therapist perspectives on barriers and facilitators for implementation of the combination of an internet and mobile-based intervention with f2f therapy for patients with depression. Examples of facilitators were patients’ interest and motivation to participate, time savings in therapy, reduction of the treatment gap and the structure of the internet-based program guiding the treatment. Examples of barriers were therapeutic alliance burdened by technical issues, limited customizability and autonomy in decision-making, negative effects and time burdens for therapists, patient reservations and less engagement in internet- and mobile-based interventions and disease-related contraindications. Similarly, Mol et al. [10] investigated therapist experiences with BT for depression; therapists were generally satisfied with providing BT.

Beyond the treatment of depression, a more recent study examined the feasibility of blended cognitive behavioral therapy for insomnia by including interviews with therapists [11]. Therapists felt supported by the online components of the treatment. However, there were also potential barriers to implementation, such as the integration of the online materials into the f2f sessions and having to adapt the therapeutic style to BT [11]. A different study investigated therapists’ experiences with a transdiagnostic internet-based emotion regulation intervention as an add-on to outpatient psychotherapy by interviewing eight therapists about their experiences with different aspects of the treatment [12]. Regarding the blended format, almost all therapists felt that the internet-based intervention transformed f2f therapy. At the same time, most therapists also felt the internet-based intervention was not integrated enough with f2f therapy.

In contrast to the number of studies examining mental healthcare professionals’ experiences with BT in the outpatient setting, only a small number of studies have focused on the experiences of inpatient mental healthcare professionals. In general, research on the feasibility of BT for severe mental disorders is still at an early stage [7]. A survey study by Hennemann and colleagues [13] showed limited acceptance of e-mental health amongst inpatient healthcare professionals of various professional groups. However, only a small portion of the surveyed sample reported having used e-mental health interventions before, and the sample was recruited from German rehabilitation centers with different diagnostic specializations. Another study [2] examined potential benefits, barriers, and facilitators of implementing e-mental health in German psychiatric inpatient care from the perspective of inpatient mental healthcare professionals. The most frequently mentioned potential benefit was being able to provide better structured psychotherapeutic treatment. Other potential benefits included, for example, online interventions being an attractive add-on to f2f treatment and patient empowerment [2]. Potential barriers included concern that inpatients may lack the necessary capabilities to take part in online therapy, that f2f contact would be neglected and that resources would be lacking. Facilitators included technical ones such as features of the internet-based interventions, staff education and training, and sufficient functional level of patients. More than three-quarters (i.e., 77%) of participants indicated having very little previous experience with online therapies [2].

Dorow and colleagues [14] interviewed (paper-pencil) 31 medical experts about their experiences with implementing an internet-based self-management tool for treating depression in psychiatric inpatient care. These professionals had a positive attitude toward the intervention. The majority stated that the intervention could be easily integrated into inpatient clinical workflows. The most frequently mentioned barriers to implementation were patient older age, difficulties with concentration, severe courses of depression and a lack of available computers.

Finally, in another study [15], psychiatric inpatient healthcare professionals in Belgium reported on their experiences with the platform Moodbuster. These professionals used the platform as an add-on to treatment as usual as they saw fit. No treatment protocol was provided. The participants valued using e-mental health applications for bridging the transition from inpatient to outpatient treatment. However, implementation was also challenging due to technical difficulties and inpatient care related factors such as lack of structural facilities. Further reported barriers were platform use not being supported by the whole team, a lack of time resources, a lack of patient interest, and short patient stays that made it difficult for patients to become familiar with the platform.

In sum, while initial findings from studies on mental healthcare professionals’ experiences point to the potential of inpatient BT, certain factors may be important to consider for implementation. These may include factors such as severity of a patients disorder, support from the multi-professional treatment team, availability of resources on the ward or duration of patient stay [14, 15]. However, experiences of mental healthcare professionals may also differ depending on the exact nature of the inpatient setting in question. For example, experiences with online interventions on a psychotherapy ward, where treatment takes several weeks and follows a set structure, may differ from experiences on an acute psychiatric inpatient ward where patients may arrive in states of crisis and turnaround is high.

Thus, the present study aimed to examine healthcare professionals’ experiences with an internet-based emotion regulation intervention added to acute psychiatric inpatient treatment. The qualitative analysis was part of the Acute-REMOTION project (NCT04990674 [16], which examines the feasibility and preliminary evidence for effectiveness of an internet-based emotion regulation intervention provided as an add-on to acute psychiatric inpatient care. By using a thematic analysis approach [17, 18] semi-structured interviews with physicians, psychologists, nursing professionals, social workers and occupational therapists were analyzed regarding their experiences with the intervention added to acute psychiatric inpatient treatment.

## Methods

### Trial and participants

Healthcare professionals’ data was collected as part of a randomized controlled pilot trial [16]. The full trial was registered with Clinicaltrials.gov (ID NCT04990674) on 04/08/2021. The purpose of the trial was to examine a transdiagnostic internet-based emotion regulation intervention as an add-on to acute psychiatric inpatient care. An intervention group (*n* = 30) that received acute psychiatric inpatient care (treatment as usual, TAU) plus access to the internet-based intervention was compared to a group that received TAU (*n* = 30). Outcomes were assessed at baseline, after 4 weeks, after 8 weeks and at inpatient discharge. A version of the internet-based intervention, REMOTION, developed at the University of Bern by LLB and TB with input from FM, had also been examined previously in the outpatient setting [19]. The web-based, smartphone compatible intervention consists of six modules and teaches patients different emotion regulation skills by including text, audio and video material along with different exercises. A detailed description of the intervention content and delivery can be found in the study protocol [16]. Data collection for the Acute-REMOTION randomized controlled pilot trial was complete in July 2023.

Healthcare professionals from three acute psychiatric inpatient wards that participated in the trial, were asked about interview participation f2f and per email. Using a purposive-convenience sampling approach, on each ward, all healthcare professionals from the following occupational groups were asked about interview participation, with the goal of interviewing a minimum of one healthcare professional per occupational group: physicians, nurses, psychologists, social workers, and occupational therapists. If more than one healthcare professional per occupational group expressed interest in interview participation, we interviewed all of the interested parties. All interviewees had completed a workshop on the internet-based intervention at trial commencement and had been given access to the internet-based intervention for after the workshop as has been done in previous studies [2]. The 20 interviewed participants were on average 36.8 years old (range 24–58). Twelve participants were female. Details about the sample are presented in Table 1. All interview participants gave written informed consent. Participants received no financial compensation for taking part in the study.

**Table 1:** Sociodemographic characteristics of participants

Full sample *N* = 20			
		*n*	%
Age			
*M (SD)*	36.8 (10.74)		
Min – Max Gender	24–58		
Female		12	60.0
Male		8	40.0
Professional group			
Occupational therapist		2	10.0
Nurse		7	35.0
Physician		4	20.0
Psychologist		5	25.0
Social worker		2	10.0
In Training^a^		4	20.0
Employment			
Full time		11	55.0
Part time		9	45.0
Work experience (psychiatric setting) in years (*M* (SD))	6.1 (6.68)		
Number of REMOTION modules processed independently after workshop participation			
None		9	45.0
One		4	20.0
Two		1	5.0
Three		1	5.0
Four		1	5.0
Six		4	20.0
Previous experience with internet-based interventions			
None		9	45.0
Little		11	55.0
Type of previous experience with internet-based intervention^b^			
No specification		3	23.1
Therapeutic context		4	30.8
Lecture/seminar		3	23.1
Research		1	7.7
Personal use		2	15.4
Internet use per day			
1–3 h per day		11	55.0
3–6 h per day		4	20.0
> 6 h per day		4	20.0
No use		1	5.0

Note. ^a^In training refers to being in a specific training that pertains to a specific occupational group (for example a psychologist in traing to become a psychotherapist). ^b^ Multiple responses possible per participant

### Semi-structured interviews and data collection procedure

Authors LLB and AL developed the semi-structured interview guide for this study. The interview guide questions were based on an interview guide used in a previous studybased on an interview guide used in a previous study [12] and complemented by elements provided in a publication by Sander et al. [2]. All interviews were conducted f2f by authors GW, LKD and AL at the University Psychiatric Services, Bern (UPD) in November 2021 and audio recorded. Interview duration was 26.3 min on average with a range of 11.4 to 50.3 min. Before conducting the interviews with the ward teams, the interview was pilot tested with a healthcare professional form a separate acute inpatient ward at the UPD. All interviewers took part in an in-house interview training session with author LLB. For each interview, the interviewer introduced herself and the goals of the overall project and specifically the interview. Prompts were provided after each interview question if necessary (see Supplementary Material 1 for an overview of the interview questions). The interviews were transcribed by authors AL, EvK and by Raël Brechbühler using transcription guidelines specified by Dresing and Pehl [20].

Interview participants were also asked to fill out a short paper-pencil questionnaire regarding demographic information at the beginning of each interview. Items on the questionnaire included the following topics: age, gender, profession, employment, being in training or not, work experience, previous experience with internet-based interventions, amount of internet use per day and number of modules processed in REMOTION.

### Data analysis

The interviews were analyzed using a thematic analysis approach [17, 18], chosen for its flexibility regarding the possibility of an inductively developed analysis that captures both semantic and latent meaning and due to its successful previous use in studies on BT and internet-based interventions [10, 21]. The analysis followed the following phases [17]:

1. LLB familiarized herself with the data; read all 20 transcripts, listened to the audio-recordings and re-read the transcripts.
2. LLB coded all 20 transcripts. The first rounds of coding produced 210 codes. These codes were then discussed with author EvK and AL who also read all 20 transcripts. Initial codes were adapted accordingly. The final set of codes included 146 individual codes.
3. Initial subthemes and themes were generated from the final set of codes by LLB.
4. Subthemes and themes were developed and reviewed with four members of the research team (EvK, AL, GW and TB) and continually revised and refined in an iterative process.
5. Five final themes were defined and named.

All qualitative analyses were conducted with the software MAXQDA 2022 [22]. All other quantitative analyses were conducted with SPSS version 29. The COREQ checklist [23] was used to ensure methodological quality. For the results section, illustrative quotes were selected to portray the essence of the subthemes. Quotes were translated from German to English for the purpose of the manuscript by LLB. When subthemes are discussed, expressions common to qualitative analytic reports [24] were used to discuss prevalence across the data. In the results section the expression *occasionally* or *a few* refers to less than half of the participants. *Several* refers to 10–13 participants. *Many*, *commonly* or *often* refers to at least 14 participants. *Almost all* refers to 18 or more participants.

### Personal relationship to the data

The first author LLB is a postdoctoral researcher and psychotherapist with experience conducting BT research and with experience in qualitative methodology. Second author GW is a doctoral student with experience with BT research and qualitative methodology. AL is a doctoral student currently working with a mixed methods research design in the field of medical education and palliative care. Previously, AL analyzed interview data of one acute inpatient ward as part of her master’s thesis. Authors EvK and LKD were master students in psychology during study conduction, EvK also has a professional background in nursing. GW, LKD and AL who conducted the interviews were integral parts of the research team for the trial, for example they were also part of the interdisciplinary ward meetings for the purpose of the trial, but they had no other relationship with the interviewees. Authors CN and FM are chief physician and chief psychologist, respectively, and both experts in the field of acute psychiatric treatment research. Author TB is a leading expert in the field of internet interventions and BT research and has expertise with qualitative analyses in this specific research area.

## Results

The following paragraphs describe the five main themes. The corresponding subthemes for each main theme are also listed with examples. An overview of the themes and subthemes can be found in Table 2. Additionally, a table showing subtheme frequencies for different occupational groups (nurses, psychologists and physicians) can be found in Supplementary Material 3.

### Lack of experience

The theme lack of experience reflects how participants had little hands-on experience with BT both prior to trial commencement and during the trial. Three overarching subthemes were identified. Regarding previous experiences, many participants reported having *few or no previous experience* using internet-based interventions during psychiatric treatment with their patients. For example, one participant said, “With such internet-based interventions, actually none at all” (9607). Others reported having theoretical knowledge on the topic, knowledge gained from the initial workshop on the internet-based intervention that was part of the trial, but not having had practical experience e.g., “regarding practical application none [experiences] at all, exactly, however we participated in the workshop” (4721). Along these lines, a few participants also reported having *no expectations* toward this type of intervention, one participant said: “I actually had no expectations at all” (9562). Interestingly, participants often reported having *few points of contact* with the intervention during actual study conduction. They reported hearing about the study from the study team, but not having had much interaction with patients who used the intervention, as described by one participant in the following way:

I had few experiences with the intervention, I don’t really know which patients took part. I was aware of you [study team] being a part of the interdisciplinary meetings and how you asked if patients could take part in the study. What happened after that, I really didn’t notice much about that, more about organizational things… (9087).

### The intervention as a contemporary complement

This theme summarily reflects the positive attitude of acute inpatient treatment providers towards an internet-based intervention added to acute inpatient psychiatric treatment. Many participants reported *positive expectations*. For example, one participant said “When I heard about it [the intervention], I thought this will briefly and intensively contribute to recovery of patients” (9087). Another example of a mentioned expectation was the patients’ improved ability to deal with emotions: “So when I saw this program, I thought okay, this is a very good assistance for our patients to better notice and express their emotions, so an additional form of help. That was my expectation” (8263). Another example comes from a participant who mentioned the potential complementary value of the intervention for transfer into daily life - “Well, my expectation was that this internet-based program alongside psychotherapy would allow for more transfer into daily life, also outside of therapy sessions, that patients can work on content, and receive a tool that complements treatment” (1584).

An internet-based intervention added to acute psychiatric inpatient treatment was described as *necessary and contemporary* by all participants in the study, reflecting the positive basic attitude regarding this type of treatment. For example, one participant said: “I think it’s enriching, absolutely. I can’t see anything negative about it right now” (3253). Along these lines, another participant said: “Yes this is the future and if we don’t jump on this, we are losing something very important” (9087). The importance of this type of treatment, specifically for future generations, was highlighted: “Mhm, so I feel very positive about this because I see that the system we live in is dependent on electronic systems, be it smartphones or tablets and the young generation only knows this and we have to speak their language, so I am actually very positive and am also prepared to use and internalize this as an instrument” (4721).

Moreover, *positive effects on therapeutic work and on patients* were described by all participants. For example, many interviewees mentioned the intervention being a complement to other acute inpatient treatment elements. One participant mentioned the complementary nature in the following way:

I just find it exciting that it is a different form with a different medium for once. I think some patients are very responsive to doing something different than always going to a group or talking to a specialist. I think also doing something on the computer for once is certainly a good thing, it’s so relieving for some (9087).

Another example is that many participants mentioned benefits of the transdiagnostic focus on emotion regulation being broadly applicable, as described by one participant in the following way “I definitely see an advantage in the fact that a broad group of people or people with a broad group of disorders was able to benefit” (7786). Another participant mentioned the intervention having a can-opener effect for conversations between patients and ward staff: “That it can foster awareness of emotions and it seemed like it was a type of can-opener, for conversations, that it kind of helped with the building of the therapeutic relationship” (5903). Someone else mentioned more detailed benefits of the content for their own therapeutic work:

Yes, for me it was positive that I was more sensitive to people, to their emotions, so I thought about whether a person would be a participant for the intervention when they entered treatment, and I then scanned them better. And then, of course, I approached these people more (7786).

Another example stems from participants mentioning patients being able to work more flexibly with the intervention, for example at their own pace. One participant said: “you can access it all the time, otherwise patients may have to wait until they get their first therapy session and this is a program which can be used immediately after intake, when the moment is right” (6772). Regarding a patient’s treatment path, participants also mentioned the benefit of continued treatment with an internet-based intervention; treatment that is accessible to patients beyond the duration of an acute inpatient stay.

The subtheme *characteristics of the internet-based program* with content mentioned by almost all participants, reflects specific aspects of the internet-based program that participants liked. These included aspects concerning the content and structure, the technology and multimedia used, the design of the program, its usability and the language used in the program. For example, one participant mentioned the structure of the program, it’s breakdown into short modules as suitable for acute psychiatric inpatients:

Yes, I think that many things are very self-explanatory, so the whole educational part is formulated in very plain language. I believe that if you are cognitively fit to some extent and your concentration is already at a level where you can concentrate quite well, then it’s really easy to understand that you can actually stop [the program] at any point. And then you don’t break off in the middle of a sentence, so to speak, but you can really look at a section and then say okay, it’s too much for me now. And then you know where you were. I think that’s very helpful because, in principle, that’s exactly what you need in the inpatient area, that you can really just look at it a little bit at a time (3771).

Another participant mentioned the language of the internet-based program in the following specific way: “Yes, the language was kept plain, I think. That’s also a positive thing. It makes it easier to stick with the program” (5778).

### Concerns about fit for acute psychiatric inpatient care

The third theme relates to concerns mentioned by participants both regarding the fit for acute psychiatric inpatients and regarding doubts about the implementation in acute psychiatric inpatient care. Regarding the subtheme *fit for acute psychiatric inpatients* with content mentioned by many participants, different concerns about adding an internet-based intervention to acute inpatient care due to fit for acute psychiatric inpatients were detailed. For example, participants mentioned the acuteness and severity of illness as obstacles, for example the state of crisis in which patients come to the ward or their inability to concentrate. One participant described the state of patients coming into acute psychiatric care as follows: “So of course, in what condition does someone come to the ward? The acute state of the patient, or often it is about stabilization. This can then be an obstacle. That they are incapable of staying focused, somehow” (3253). Another example regards a lack of affinity towards technology as a patient-specific barrier as summarized by the same participant: “Maybe another aspect - that the people who aren’t used to it [technology], that they have a certain barrier «what now I also need to install this program!?» Exactly, the technological aspect. That, yes” (3253).

The subtheme *doubts about implementation*, with content mentioned by several participants, details concerns about the implementation process. For example, a few participants did not want an internet-based intervention to replace parts of or completely, f2f treatment. One participant said “What one can see negatively is that people will spend even more time with their technological tools and less with their therapists, with other patients or with nurses” (5778). Another example is that participants felt the intervention may be better suited for a different inpatient setting. One participant said:

So generally I think this is a great idea. It may be difficult to implement on this specific ward due to various reasons that were already mentioned. Yes, I think on a different ward, or maybe less acute ward this would definitely be very valuable (1584).

Along those lines someone else said:

It could also be that they [the patients] feel a bit outsourced. In the sense of, “do your program yourself”. You come here because you have the feeling that you can’t do it yourself, you need help from the outside. And then you’re given something to do on your own, again. Of course, it is supposed to help you, but is it understood that way? (9607).

Several prerequisites for successful implementation were also mentioned by participants. These prerequisites are touched upon in the next two themes.

### The human factor as essential for implementation

This theme touches on several facets of the human factor that are important for implementing an internet-based intervention added to acute inpatient psychiatric treatment. On the one hand, the first subtheme, *the team makes or breaks it*, with content mentioned by almost all participants, highlights different examples of how the team is essential for implementation. For example, the importance of the ward team having insight into the intervention, being involved in it, and supporting the intervention was mentioned. One participant said:

That they [the employees] know what patients are actually doing, that they know the exercises, that they know the benefits of them, but that they can also say “Okay, maybe today isn’t the right day to deal with your emotions if things are already very difficult” To be able to cushion things and so on. I think you definitely have to get the employees on board (3771).

Along these lines, participants mentioned the importance of having someone be responsible for the intervention on each ward and the importance of having someone checking patient eligibility:

That there is a fixed scheduled procedure, for which there is also a responsible person on the ward. That means that for an internet-based program or for anything that is scalable and easily accessible, there needs to be a responsible person per ward that thinks, is this useful for the patients that we admitted in the past days? A specific starting point, a specific contact person and that there is this feeling of responsibility for the execution of the program. Because otherwise it’s so free floating, no one really feels responsible. No one feels properly responsible for it, and then at some point it is, this is a personal experience that can of course also be wrong. That it’s like, that one thinks, oh for difficult patients where nothing else works, oh yes, there is also REMOTION, we could try that (5903).

Another example comes from a few participants mentioning the importance of support from leadership and management, as detailed by one participant, “No, I think it really needs to be from the side of management, leadership or whatever, room for this type of treatment should be established” (9607).

Moreover, human guidance for patients was commonly described as important (subtheme *guidance is key*). For example, one participant said “Yes, I think this is important, that they don’t just work with the program but are also guided through it” (6772). Another participant said “In the acute setting it is difficult for our patients. Using REMOTION consequently, I noticed that they needed support from us” (7786). Regarding examples of how exactly this guidance should be provided, an introduction to the internet-based program was mentioned:

Mhm. Yes, or I believe what I could also imagine is that when psychologists on the ward introduce patients to the program, that they also provide an introduction. I don’t think a group format would make more sense. But maybe an individual introduction for patients that are suitable. Yes, something like that (5778).

Other suggestions included, for example, the sending of reminders within the internet-based program by therapists, technical support or a peer support function.

Finally, many participants identified specific patient characteristics as important for implementation **(**subtheme *patient characteristics*). The most frequently mentioned patient characteristic was motivation. One participant said:

Being motivated. Yes. If they feel forced to do it, they won’t do it. Too much pressure is, one has to, the intrinsic motivation needs to be fostered for them to do it, for them to enjoy it, to voluntarily do it (7659).

Similarly, another participant said “They need to bring a kind of motivation to, to be ready at all, to work on it themselves, and not to participate in programs where everything is predetermined for them, something like that” (3253). Various other characteristics were also mentioned, including, for example, cognitive prerequisites such as being able to concentrate, or being independent and self-disciplined, as detailed in a statement from one participant: “So I think you have to have a certain amount of self-discipline, and if you have that, then it can, it is certainly only positive” (5778). A further aspect discussed as relevant to implementation was patient diagnosis. A few participants wished a broadening of inclusion criteria for the study and felt the intervention was suited to a broader range of diagnoses. For example, one participant said “The thing that bothered me a bit is that I sometimes thought that it would also have been good for people with mild psychotic symptoms.” (7786).

### Requirements for implementation beyond the human factor

Aside from the human factor, other specific requirements for successful implementation were mentioned. On the one hand, *integration into existing treatment structures* was occasionally deemed important by participants. If the internet-based intervention is not integrated well into existing treatment structures, it may become a burden. One participant said “If this just runs alongside treatment, it is an additional burden. And everyone knows, additional burdens will be cast aside, and one starts concentrating on the essential things” (9607). Specific ways to better integrate the internet-based program into the acute treatment were mentioned by participants such as providing a fixed program slot for the intervention, allowing the intervention to be prescribed during a stay or integrating the intervention into the clinic-wide IT-system. Regarding a slot in the ward program one participant said:

“If there was a fixed time, reminding the patient every morning at eight o’clock that the study is taking place, then this would be a fixed point” (9607).

Concerning implementation in the clinic-wide IT system another participant said:

So I’ll repeat myself here, I have the feeling that it [the intervention] wasn’t that integrated. For example, that, I mean, we all work via our digital clinic system, that it’s [the intervention] somehow noted there. In the ideal case, you would make a brief note, or the program would automatically note which exercises the patient has just completed and, I haven’t seen this… (626).

Another factor that was often deemed necessary for successful implementation was the availability of *resources*. These included technology on the ward, spatial resources, financial resources, and personnel capacities. For example, one participant said:

I believe that we have good internet everywhere, which is the basic requirement for this. But I don’t think that’s the biggest problem in the clinic. What may be a problem is that not all patients have a device where they can use it [the internet-based program]. There is a PC for the patients. But it’s in the corridor. That’s not exactly ideal in terms of privacy. And especially if you’re perhaps doing things that make you a bit emotionally preoccupied AND you have difficulties regulating your emotions, it might not be ideal in the corridor (3771).

Another participant mentioned personnel capacities as lacking for implementation:

It’s a bit like that, a lack of exchange due to a lack of time somehow. The fact that everything has to work somehow, has to happen quickly, that yes, capacities are lacking, a little bit. To give it [the intervention] weight, yes (3253).

Specific *changes to the internet-based program* were also suggested by almost all participants including aspects regarding technical aspects, language, content and structure of the program, the amount of information in the program and the available evidence-base. Several participants reported the desire for technical changes such as the availability of the intervention as a native app. Easy to understand language and the availability in other languages than German were also considered important by several participants. For example, one participant said:

We also have many patients who don’t speak German and then I’ve also heard that it might be cool if there were several languages but there’s also the problem that they could then be accompanied less then and that’s also difficult (6772).

Finally, the *timing* of the intervention within an acute inpatient stay was often considered important for implementation and acute psychiatric inpatient treatment was described as hectic and unpredictable by participants.

But then there’s also the unpredictability of our acute ward, if you’re in a specific ward program, for example, there’s a clear setting - that’s how many weeks you’re likely to be there. Here, they may be here today and gone tomorrow. And if I want to implement something and don’t know whether the patient will be gone again tomorrow, it’s really difficult (9607).

Giving patients enough time to reach a certain level of stability during the acute inpatient stay but then also not waiting too long with intervention use because patient stays are short seemed to be a relevant factor to consider for implementation. For example, one participant said:

What I would definitely do is open up the time span a bit. Give people a little more time so that they don’t have to go so deep in the first nine to ten days after entering treatment. That is certainly something I would like to see implemented (7786).

Another participant said “and, or perhaps still, we asked them directly as soon as the person arrived, and we realized that they first had to orient themselves a bit…” (1093), alluding to the fact that patients may not be able to start with this type of intervention from the get go of an acute inpatient stay.

**Table 2:** Overview of the five main themes and corresponding subthemes

Theme and subthemes	
**Lack of experience** Few or no previous experiences No expectations Few points of contact **The intervention as a contemporary complement** Positive expectations Necessary and contemporary Positive effects on therapeutic work and patients Characteristics of the internet-based program **Concerns about fit for acute psychiatric inpatient care** Fit for acute psychiatric inpatients Doubts about implementation **The human factor as essential for implementation** The team makes or breaks it Guidance is key Patient characteristics **Requirements for implementation beyond the human factor** Integration into existing treatment structure Resources Changes to the internet-based program Timing	

Note. Main themes in bold

## Discussion

This study intends to provide insight to mental healthcare professionals’ experiences with an internet-based intervention provided as an add-on to acute psychiatric inpatient treatment. Five overarching themes and corresponding subthemes were identified. First, a lack of experience with this type of intervention, both prior to and during the study, was described by the interviewees. This theme resonates with previous research showing the lack of experience with digital interventions in psychiatric inpatient care [2, 13, 15]. Interestingly, the current study was conducted after the start of the Covid-19 pandemic. Still, individuals reported few contact points with BT. This may be explained by the fact that a more common form of digital intervention that was utilized during the pandemic was psychotherapy per videoconferencing, not necessarily an internet-based intervention added to f2f treatment.

The second theme focused on the positive potential of adding an internet-based intervention to acute psychiatric inpatient treatment. Health professionals saw the intervention as a contemporary complement to acute inpatient psychiatric treatment. Participants felt this was a modern treatment option suited especially to future generations. This finding is in line with a study by Dorow et al. [14], where the internet-based intervention was deemed suitable to younger patients by participants. Moreover, positive effects on therapeutic work and on patients were reported in the interviews of our study. For example, according to participants, the transdiagnostic emotion regulation focus of the intervention was considered valuable for acute inpatient treatment. Future research should focus on the feasibility and efficacy of transdiagnostic interventions in this specific setting. One example of a feasibility study has been provided by Bendig et al. [25].

The third theme focused on concerns about the fit for an acute psychiatric inpatient care setting. Similarity to previous research on inpatient settings [14], interviewees mentioned concern about this type of intervention perhaps not being suited for more severely ill patients. Specifically, concern about certain disorders, acute phases of certain disorders, individuals in severe crises and with a lack of stability at the beginning of an acute inpatient stay were mentioned. Along these lines, Sander and colleagues [2] state that patients lack of capability due to symptom severity or cognitive incapabilities, is a potential barrier to implementing online therapy in psychiatry inpatient care. Moreover, doubts about implementation of internet-based interventions in acute psychiatric inpatient care were also mentioned in our conducted interviews, for example that internet-based interventions should not replace f2f treatment.

The final two themes focused on factors relevant for successful implementation in acute psychiatric inpatient care. On the one hand, the importance of what we deemed the human factor was revealed. This resonates with the recent proposition by Westheimer and colleagues [26] regarding the importance of what they term having both high-tech and high-touch protocols for digital interventions in inpatient settings. It is also in line with what Ehrt-Schäfer et al. [7] term the need for human facilitation of digital interventions for severe mental disorders. In our study the support and involvement from the ward team was deemed important. This is comparable to results from the study by van Assche et al. [15], who mentioned the lack of an implementation strategy being carried by the entire team leading to fragmented use of their internet-based intervention. Furthermore, human guidance was viewed as important by participants in our study, both f2f and within the internet-based program. This is interesting since the intervention already included f2f contact with the study team. Interviewees seemed to support additional human guidance specifically provided by members of the ward team, again alluding to the importance of support from within the ward. Moreover, certain patient characteristics were seen as important for implementation including, for example, patient motivation. Interestingly, a few participants also reported that they would have liked to see a broader implementation of the intervention to a wider range of patient diagnoses. For whom in acute inpatient care transdiagnostic BT focused on emotion regulation is specifically suited, should be examined in a future study that analyses quantitative data from a larger sample.

Beyond the human factor, aspects such as better integration of the internet-based intervention into existing treatment structures, the sufficient availability of financial, technological, and spatial resources, and timing of the intervention within acute psychiatric care were deemed relevant factors for successful implementation. Regarding integration, Sander [2] mention that internet-delivered interventions should be highly interoperable with existing digital documentation systems and be integrated into the inpatient workflow. Along these lines, Liu and Schueller [27] describe how digital mental health interventions that align with existing workflows, clinical guidelines, and routines are more likely to be successfully implemented. In our study, professionals recommended not treating patients with this type of intervention too early during an acute stay. At the same time, inpatient stays are often short, making it difficult to get used to a new e-mental health interventions in an adequate amount of time [15]. Timing (not to early, but also not too late) may therefore be a delicate and important factor to consider for the success of BT, where an internet-based intervention is meant to be applied as an add-on to acute psychiatry inpatient care.

Inpatient implementation of digital interventions has been aptly described as a complex interplay of a wide range of factors at different levels [15]. The same can be said for implementation in acute psychiatric inpatient care. Treatment in this setting may be characterized by specific elements such as patient’s symptom severity, urgency of care and fast turnaround times. The fact that most healthcare professionals noted positive aspects of this type of intervention for acute inpatient psychiatric treatment, despite the potential barriers, makes future research on the successful implementation of internet-based interventions added to acute psychiatric inpatient treatment especially valuable. The following paragraphs provide tentative recommendations for the implementation in this setting.

### Recommendations for future implementation

**Fig. 1:**
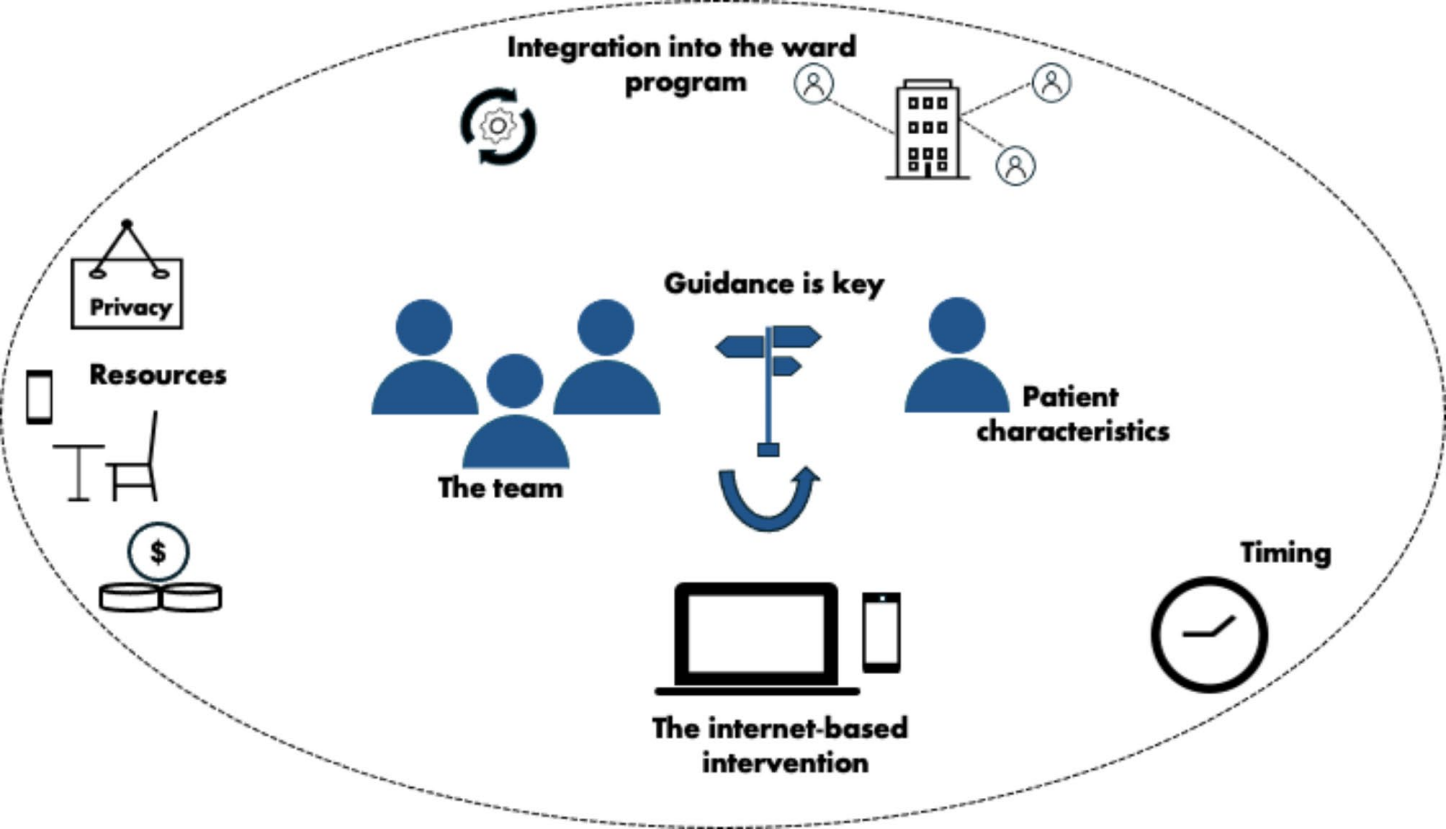
Factors to consider for implementation Note. Factors to consider for implementation based on themes four and five of the thematic analysis. Subthemes that belong to *the human factor as essential for implementation* are portrayed in blue

According to a recent review, BT holds promise for effective, potentially cost-effective, and more accessible treatment for patients with severe mental health disorders [7]. The results of the current study, specifically from themes four and five, can be used to make tentative recommendations to assist future implementation of transdiagnostic internet-based interventions added to acute psychiatric inpatient care based on healthcare professionals’ experiences. The recommendations are summarized in Fig. 1 and highlight the interplay between patient, multi-professional team, and the internet-based intervention, embedded in the acute inpatient psychiatric setting.

Based on theme four, the human factor as essential for implementation, involvement and support from the treatment team is important. Support can be enhanced by informing the entire team about BT, granting access to the online program and patient progress, and defining team roles regarding BT clearly. Perhaps, similar to what Schueller & Torous [28] term internal champions, individuals who have the proper training and also integrate discussions of BT into meetings with the team, could help in this regard. Another important aspect for implementation may be the provision of human guidance for patients. This may be especially important at the beginning of BT where patients are provided with a rationale for BT and where they are provided with an introduction to the use of the internet-based technology. Regarding patient characteristics, patient motivation may be fostered by including a motivational module as part of the internet-based intervention. Also, an intervention similar to an acceptance-facilitating intervention examined by Kreis et al. [29] could complement the implementation process. Additionally, eligibility for transdiagnostic BT in inpatient settings may be better determined by factors like emotion regulation difficulties, assessed through brief, setting-appropriate questionnaires [30], rather than by diagnosis alone.

Based on theme five, integrating the intervention into existing treatment structures may want to be considered for successful implementation. This could include assigning a fixed slot in the ward treatment plan and incorporating BT as a treatment option in the clinic’s IT system, allowing healthcare professionals to monitor progress and prescribe BT, similar to medication. The prescription of digital therapeutics is already becoming increasingly popular in other countries around the world [31]. Regarding ward resources, necessary technology and a private space for patient use should also be provided. Additionally, offering the program in multiple languages and eventually as a native app could improve accessibility. Finally, the timing of BT within acute inpatient treatment may want to be considered. BT may not suit all patients immediately upon intake, especially those in crisis. Starting BT during the inpatient stay with an option to continue post-discharge could help provide continuity in the treatment process.

### Strengths and limitations

To our knowledge, this is the first study that examined in detail the experiences of healthcare professionals with a transdiagnostic internet-based emotion regulation intervention added to acute psychiatric inpatient treatment via analysis of interview data. Unlike other studies, all healthcare professionals interviewed in this study had exposure to the intervention, on the one hand through a workshop conducted at the start of the main trial and on the other hand through study participation over several months on their designated wards before interview conduction. Moreover, a broad range of occupational groups were interviewed, reflecting the interdisciplinary nature of acute psychiatric inpatient care. This study also has several limitations. First, only 20 individuals from three wards were interviewed with an imbalance between occupational groups. It is important to reiterate that no inferences can be drawn about the prevalence of phenomena observed beyond the specific sample. Second, some participants reported having limited contact with the patients who were randomized into the intervention group, and nine participants reported not having individually processed REMOTION module content. Future studies may want to conduct the interviews at an even later date during trial conduction. Third, only the perspective of healthcare professionals was examined within this study. Future studies should aim to also include the perspective of the patients and compare the two. Finally, the experiences described are specific to a form of BT where an internet-based intervention was provided as an add-on to inpatient psychiatric treatment without a high degree of integration between f2f and internet-based elements. Future research should examine healthcare professionals’ experiences with different forms of BT in the acute inpatient psychiatric setting.

## Conclusions

The results of the study indicate that an internet-based intervention is deemed a contemporary complement to other elements of acute psychiatric inpatient treatment by healthcare professionals who work in this setting. Interviewees described the potential to improve acute inpatient treatment with this type of intervention. At the same time, concerns about fit for acute psychiatric inpatient treatment and doubts about implementation remain. For successful implementation of BT in this treatment setting, certain factors may be especially important. On the one hand, human factors seem to play an important role, on the other hand, elements such as integration into existing treatment structures, resource availability, and timing of BT within a patient’s acute psychiatric inpatient treatment may be of relevance.

## Electronic supplementary material

Below is the link to the electronic supplementary material.

Supplementary Material 1

Supplementary Material 2

Supplementary Material 3

## Data Availability

The raw datasets analyzed during the current study contain sensitive data that cannot be made publicly available. A dataset without sensitive information is available from the corresponding author upon reasonable request.

## Abbreviations

f2f: Face-to-face
BT: Blended treatment
TAU: Treatment as usual
UPD: University Psychiatric Services, Bern, Switzerland
