# Supplementary material for: A qualitative analysis of healthcare professionals’ experiences with an internet-based emotion regulation intervention added to acute psychiatric inpatient care
Source: BMC Psychiatry. 2024 Dec 27;24:955. doi: 10.1186/s12888-024-06365-z (PMC11674089; doi:10.1186/s12888-024-06365-z)
Supplement: Supplementary file 1 — Supplementary Material 1 [file 12888_2024_6365_MOESM1_ESM.docx]

**Additional File 1:** Interview guide (translated from German into English)

***Introduction***

- My name is ____, I am part of the study team of the Acute-REMOTION project
- As already announced, as part of the Acute Remotion Project, I will conduct this interview with you. Thank you very much for your participation!
- First I will inform you about the interview and the procedure and then I will start with the interview questions.
- I will now start with the information regarding the interview.
- Is that okay with you?
- I would like to draw your attention once again to the fact that this interview will be recorded (audio-recording). The recording will then be transcribed and the audio-recording deleted after transcription & study completion.
- The transcripts are then analyzed further for publication purposes. Any potential publication does not contain any information that makes you identifiable as an individual.
- With this interview we would like to know more about your experiences (satisfaction, attitudes etc.) with the blended intervention (REMOTION + acute inpatient treatment).
- We are particularly interested in what you liked or disliked about the intervention or what should be adapted for better implementation in the acute inpatient treatment setting.
- I will ask you several open-ended questions.
- The interview will take about 30-45 minutes
- You can ask me questions at any time if anything is unclear.
- Please answer openly and freely.
- We will conduct the interview in German, but if any Swiss German words come to mind in between answers, this is not a problem.
- *Instruction to the interviewer: obtain verbal consent for interview participation.*
- Do you have any further questions? Now we’ll start with the interview questions.

**Interview questions:** *Instruction to the interviewer: If the person only answers one aspect of a question (e.g. only says what was helpful) repeat the second aspect of the question - e.g. "and what else was perhaps bothersome".*

1. **Expectation / Motivation**
   - What were your expectations toward the intervention (REMOTION + acute inpatient treatment)?
   - To what extent have you already had experience with such interventions?
2. **General experience / impression**
   - How did you experience the intervention (REMOTION + acute inpatient treatment) in general?
   - Which aspects of it were helpful, bothersome or lacking for you?
   - What was helpful, bothersome or lacking about the intervention (REMOTION + acute inpatient treatment) compared to normal acute inpatient treatment?
   - What do you think was the advantage or disadvantage of the intervention not targeting specific mental illnesses, but targeting a transdiagnostic construct: the regulation of emotions?
3. **Format**
   - How did you feel about the way the internet-based program was integrated into acute inpatient treatment? Which aspects of it were helpful, bothersome or lacking?
4. **Internet-based program**
   - How did you experience the internet-based program (design, language, structure, content)? Which aspects were helpful, bothersome or lacking for you?
5. **Impact**
   - Were you able to see effects of the intervention on patients? If yes, what kind of effects (positive and negative)?
   - Did the intervention have an impact on your relationship with patients?
   - Were you able to determine the influence of the intervention on your own work? If so, what kind of influence (positive and negative)?
6. **Implementation**

- What is needed for the successful implementation of such an intervention in the acute inpatient setting?
- What is specifically needed:
  - - In terms of technology?
    - With regard to patient characteristics?
    - With regard to organizational factors?
- What are possible obstacles to the successful implementation of such an intervention in the acute inpatient setting?
- What are possible obstacles specifically:
  - - In terms of technology?
    - With regard to patient characteristics?
    - With regard to organizational factors?

1. **Future implementation in the healthcare system**

- How do you feel about the goal of routinely integrating such interventions into our healthcare system in the future? In such a way that you can integrate and use them in your day-to-day work?

***Closing the interview****:*

- - We have now reached the end of the interview. Are there any topics that you would like to discuss that we have not yet discussed?
  - Do you have any unanswered questions?
  - Thank you very much for your participation!
