# Supplementary material for: A qualitative analysis of healthcare professionals’ experiences with an internet-based emotion regulation intervention added to acute psychiatric inpatient care
Source: BMC Psychiatry. 2024 Dec 27;24:955. doi: 10.1186/s12888-024-06365-z (PMC11674089; doi:10.1186/s12888-024-06365-z)
Supplement: Supplementary file 3 — Supplementary Material 3 [file 12888_2024_6365_MOESM3_ESM.docx]

**Additional file 3**

|  |  |  |  |
| --- | --- | --- | --- |
| **Theme** Subtheme | **Occupational group:**  **Psychologists (n = 5)** | **Nurses (n = 7)** | **Physicians (n = 4)** |
| **Lack of experience** |  |  |  |
| Few or no previous experiences | 80.0% | 71.4% | 100.0% |
| No expectations | 0 | 42.9% | 50.0% |
| Few points of contact | 80.0% | 85.7% | 100.0% |
| **The intervention as a contemporary complement** |  |  |  |
| Positive expectations | 100.0% | 71.4% | 25.0% |
| Necessary and contemporary | 100.0% | 100.0% | 100.0% |
| Positive effects on therapeutic work and patients | 100.0% | 100.0% | 100.0% |
| Characteristics of the internet-based program | 80.0% | 100.0% | 75.0% |
| **Concerns about fit for acute psychiatric inpatient care** |  |  |  |
| Fit for acute psychiatric inpatients | 80.0% | 85.7% | 100.0% |
| Doubts about implementation | 40.0% | 71.4% | 75.0% |
| **The human factor as essential for implementation** |  |  |  |
| The team makes or breaks it | 100.0% | 100.0% | 100.0% |
| Guidance is key | 60.0% | 85.7% | 50.0% |
| Patient characteristics | 80.0% | 42.9% | 75.0% |
| **Requirements for implementation beyond the human factor** |  |  |  |
| Integration into existing treatment structure | 40.0% | 42.9% | 75.0% |
| Resources | 100.0% | 85.7% | 50.0% |
| Changes to the internet-based program | 80.0% | 85.7% | 100.0% |
| Timing | 100.0% | 57.1% | 75.0% |

Table 1. Subtheme frequencies (in percent) per occupational group for psychologists, nurses and physicians.
